# Identifying Eukaryotes and Factors Influencing Their Biogeography in Drinking Water Metagenomes

**DOI:** 10.1021/acs.est.2c09010

**Published:** 2023-02-24

**Authors:** Marco Gabrielli, Zihan Dai, Vincent Delafont, Peer H. A. Timmers, Paul W. J. J. van der Wielen, Manuela Antonelli, Ameet J. Pinto

**Affiliations:** † Dipartimento di Ingegneria Civile e Ambientale—Sezione Ambientale, Politecnico di Milano, Milan 20133, Italy; ‡ Research Center for Eco-Environmental Sciences, Chinese Academy of Sciences, Beijing 100085, China; § Laboratoire Ecologie et Biologie des Interactions (EBI), Equipe Microorganismes, Hôtes, Environnements, Université de Poitiers, Poitiers 86073, France; ∥ KWR Watercycle Research Institute, 3433 PE Nieuwegein, The Netherlands; ⊥ Department of Microbiology, Radboud University, Heyendaalseweg 135, 6525 AJ Nijmegen, The Netherlands; # Laboratory of Microbiology, Wageningen University, 6700 HB Wageningen, The Netherlands; ∇ School of Civil and Environmental Engineering, Georgia Institute of Technology, Atlanta, Georgia 30332, United States

**Keywords:** drinking water microbiome, drinking water distribution systems, metagenomics, eukaryotes

## Abstract

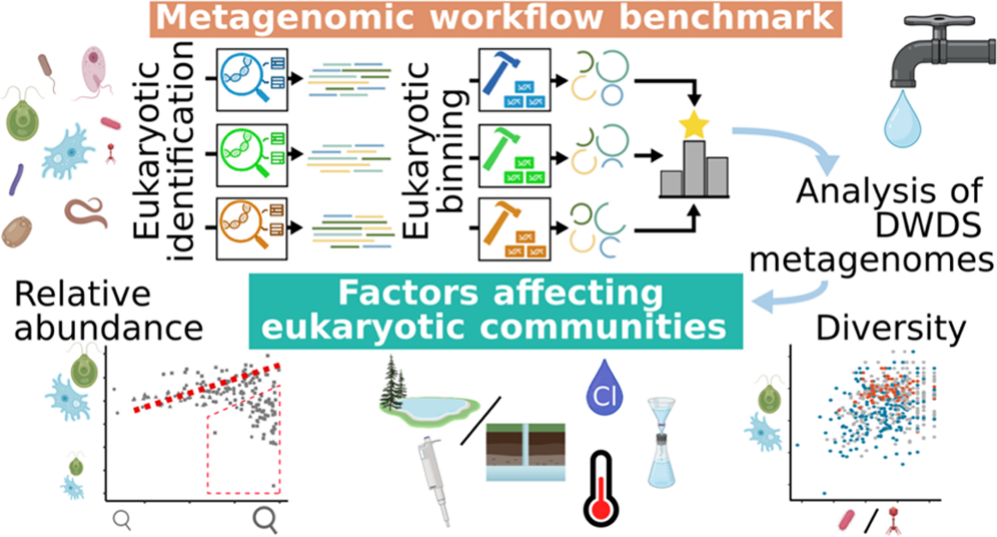

The biogeography of eukaryotes in drinking water systems is poorly understood relative to that of prokaryotes or viruses, limiting the understanding of their role and management. A challenge with studying complex eukaryotic communities is that metagenomic analysis workflows are currently not as mature as those that focus on prokaryotes or viruses. In this study, we benchmarked different strategies to recover eukaryotic sequences and genomes from metagenomic data and applied the best-performing workflow to explore the factors affecting the relative abundance and diversity of eukaryotic communities in drinking water distribution systems (DWDSs). We developed an ensemble approach exploiting *k*-mer- and reference-based strategies to improve eukaryotic sequence identification and identified MetaBAT2 as the best-performing binning approach for their clustering. Applying this workflow to the DWDS metagenomes showed that eukaryotic sequences typically constituted small proportions (i.e., <1%) of the overall metagenomic data with higher relative abundances in surface water-fed or chlorinated systems with high residuals. The α and β diversities of eukaryotes were correlated with those of prokaryotic and viral communities, highlighting the common role of environmental/management factors. Finally, a co-occurrence analysis highlighted clusters of eukaryotes whose members’ presence and abundance in DWDSs were affected by disinfection strategies, climate conditions, and source water types.

## Abstract 2

After benchmarking tools and developing a dedicated consensus workflow for eukaryotic sequence detection in metagenomes, the experimental, environmental, and engineering factors affecting their biogeography in drinking water distribution systems were investigated.

## 1 Introduction

Several drinking water regulations (e.g., refs (1−3)) include parasitic eukaryotes such as *Giardia lamblia* and *Cryptosporidium* spp. among the microbial parameters of interest due to their potential negative effects on human health.^4,5^ However, compared to prokaryotes and especially bacteria, a few studies have focused on the presence and the ecological role of unicellular and multicellular eukaryotes within drinking water distribution systems (DWDSs). These studies, employing targeted approaches (e.g., internal transcribed spacer—ITS, 18S rRNA) and traditional culture-based methods, showed that variations in the eukaryotic community in drinking water systems are associated with water quality characteristics (e.g., organic carbon, nutrients), source water type, and disinfection strategies.^6,7^ The presence of eukaryotes in drinking water systems has been shown to affect other microorganisms, for example, by shielding opportunistic pathogens from disinfection and altering biofilm properties through grazing^8^ and colonization.^9^ In addition, eukaryotes have been linked to operational issues, such as the increased presence of sediments within DWDSs and consumer complaints.^10^ For these reasons, these microorganisms should not be ignored during DWDS management but, instead, should be included in the design of ecologically-informed management practices. Leveraging knowledge of the microbial ecology of the drinking water microbiome could enable strategies to engineer drinking water microbiomes to address current operational issues and guarantee safe water at the consumers’ taps.^11^

Gene-targeted approaches (i.e., amplicon sequencing) have been recently used to not only detect a vast diversity of eukaryotes in drinking water systems but also to probe their activity in different conditions.^7^ These techniques have also revealed the widespread presence of eukaryotic pathogens in DWDSs, even in the presence of a disinfectant residual.^12,13^ However, amplicon sequencing approaches provide limited ecological and physiological insights since these may not permit fine-scale taxonomic resolution and do not provide any information on functional traits (e.g., trophic modes),^14,15^ limiting their utility in devising ecologically-informed DWDS management strategies. In addition, such insights could also be impacted by several biases arising from polymerase chain reaction (PCR) amplification, poor comparability of results between different hypervariable regions, and variable small subunit (SSU) rRNA gene copy numbers.^16,17^

In contrast, shotgun DNA sequencing (i.e., metagenomics) alleviates these limitations by directly sequencing extracted genetic material collected from the sample; this enables genome and metabolic reconstruction of the detected microorganisms,^18^ metabolic interaction inferences,^19^ and potential guiding management strategies. However, this comes with the drawback of reduced detection limits as compared to amplicon sequencing approaches.^20^ Further, metagenomic approaches can prove challenging when dealing with complex genomes such as the eukaryotic ones, especially for organisms with low relative abundances,^21,22^ such as those expected for DWDS eukaryotes. In addition, eukaryotic-focused data analysis workflows are relatively less developed compared with those targeting prokaryotes or viruses and no extensive comparison among the different options has been conducted, limiting their use to study the DWDS microbiome.

To advance the knowledge regarding eukaryotes in DWDSs, this study (i) benchmarked several approaches for eukaryotic sequence detection using synthetic metagenome constructs and then (ii) applied the optimized eukaryotic sequence detection workflow to publicly available DWDS metagenomes to characterize the diversity and biogeography of eukaryotic communities in drinking water. Specifically, we investigate (iii) experimental, environmental, and management (i.e., disinfection strategies) factors that may impact eukaryotic detection and (iv) their associations with eukaryotes, prokaryotes, and viruses.

## 2 Materials and Methods

### 2.1 Bioinformatics Tool Benchmarking

#### 2.1.1 Data Sources and In Silico Mock Metagenome Construction

The eukaryotic and prokaryotic genomes used to benchmark bioinformatics tools were downloaded from NCBI Genbank,^23^ RefSeq,^24^ and JGI Genome Portal.^25^ A data set was created including 33 eukaryotic and 216 prokaryotic genomes (Table S1). These genomes were selected after determining their absence from training sets of the *k*-mer-based tools tested in this study. To evaluate eukaryotic sequence identification tools, test contig sets were created by extracting 100 randomly selected sequences of lengths 1, 3, and 5 kbp from contigs present in the downloaded genomes, similar to previous studies.^26−29^ Benchmark samples and assemblies for eukaryotic sequence binning were obtained using CAMISIM v1.3.^30^ Specifically, 15 mock metagenomes were simulated using the genomes from Table S1, followed by generation of three metagenomic assemblies (five mock metagenomes per assembly). While all parameters were kept as default, the composition of relative genomes abundances in the different samples was drawn from a lognormal distribution (μ = 1, σ = 2), imposing a ratio of total base pairs (bp) equal to 0.05 between prokaryotic and eukaryotic reads in all samples (Table S2).

#### 2.1.2 Benchmarking Workflows for the Identification of Eukaryotic Sequences Using In Silico Mock Metagenomes

EukRep v0.6.6,^29^ Tiara v1.0.2,^27^ Whokaryote v0.0.1,^28^ and DeepMicrobeFinder^26^ were used to identify eukaryotic contigs in the generated test contigs sets using *k*-mer-based approaches. EukRep and Tiara were implemented using three different thresholds varying from lenient to stricter classifications. Majority voting identification (ties excluded) was obtained by combining the results from EukRep, Tiara, and Whokaryote, and, alternatively, Tiara, Whokaryote, and DeepMicrobeFinder to test the complementarity of the different tools. Sequences were also classified using Kaiju v1.8.2^31^ (nr_euk database version: 2021-02-24) and CAT v5.2.3^32^ (database version: 20210107), which rely on Prodigal^33^ and DIAMOND.^34^ Finally, a hybrid strategy combining the results of reference and *k*-mer-based approaches was tested. This strategy identified eukaryotic contigs using reference-based tools and then used *k*-mer-based characterizations for contigs with no available reference-based annotations. After the classification of each contig, the sequences were randomly subsampled to achieve a eukaryotic to prokaryotic bp ratio equal to 0.05, considered as a representative ratio of the relative abundance of the two superkingdoms in DWDS metagenomes^35^ to obtain performance estimates representative of real-world situations.^26^ To properly account for both false positives and negatives in imbalanced data sets,^36^ classification performances were evaluated based on the Matthews correlation coefficient (MCC), precision, and recall using yardstick v1.1.0.^37^ The subsampling was repeated 100 times, estimating the mean and standard deviation of each performance metric. A flowchart of the eukaryotic contigs identification benchmarking is shown in Figure S1.

#### 2.1.3 Benchmarking Workflows for Binning of Eukaryotic Sequences Using In Silico Mock Metagenomes

The gold standard assemblies generated by CAMISIM were classified using a hybrid reference and *k*-mer-based strategy, imposing minimum contig lengths for reference-based and *k*-mer-based classifications equal to 1 and 3 kbp based on the findings from classifier testing (see the results in Section 3.1.1), respectively. Contigs were binned with CONCOCT v1.1.0,^38^ MetaBAT2 v2.15,^39^ SemiBin v0.7.0,^40^ and VAMB v3.0.2^41^ according to the following strategies: (i) binning the full assemblies (FULL), (ii) binning only the contigs classified as eukaryotic (EUK-only), or (iii) binning contigs classified as eukaryotic and unclassified contigs (OTHER-rem) (i.e., removing contigs classified as prokaryotes or viruses). To include eukaryotic taxonomic information in SemiBin, contigs’ taxonomic assignment was performed using CAT. While all of the binners were tested using default settings, MetaBAT2 was also run with the minCV parameter equal to 0.1 and 0.33. Each strategy was tested with minimum contig length cutoffs of 1, 1.5, and 3 kbp, which corresponded respectively to the shortest default minimum contig length, the minimum contig length accepted by MetaBAT2, and a value comparable to the longest default minimum contig length. The binning quality was evaluated, focusing on the bins with the majority of the bp derived from eukaryotic genomes. Binning results were evaluated using AMBER v2.0.3^42^ on (i) the percentage of eukaryotic bp binned, (ii) the tradeoff between bin purity and completeness, assessed through the *F*1 score, and (iii) the similarity between the recovered bins and the original eukaryotic genomes, measured through the adjusted rand index (ARI). A flowchart of the procedure is presented in Figure S2.

### 2.2 Analysis of Publicly Available DWDS Metagenomes

#### 2.2.1 Data Sets Used

Metagenomes derived from DWDSs were downloaded from NCBI using SRA Toolkit v2.9.6^43^ or, if deposited on MG-RAST,^44^ retrieved directly from corresponding researchers for a total of 181 distinct samples. Only samples derived from finished water at drinking water treatment plants (DWTPs) and DWDSs were considered, excluding those either collected in raw water, within drinking water treatment plants, and DWDS biofilms. Table S3 includes a list of the samples with the details regarding experimental procedures used for each sample.^35,45−56^

#### 2.2.2 Bioinformatic Analyses

Raw reads from metagenomes were quality filtered and trimmed using fastp v0.20.1/v0.23.2,^57^ followed by vector contamination removal using the UniVec_Core database^23^ and BWA-MEM v0.7.17 or BWA-MEM2 v2.2.1,^58^ SAMtools v1.9,^59^ and bedtools v2.30.0.^60^ If a sample was sequenced over multiple sequencing runs, then the cleaned reads were merged into a single file. Cleaned reads were then used to estimate the metagenome sequencing coverage using Nonpareil v3.4.1^61^ and screened to identify the number of read pairs properly mapping to the 16S or 18S rRNA genes contained in SILVA database v138.1^62^ using PhyloFlash v3.4^63^ and SAMtools.

Cleaned reads from samples collected within each distribution system were coassembled using metaSPAdes v3.10.1/v3.15.3^64^ after filtering for contigs greater than 1 kbp by SeqKit v2.1.0.^65^ The coverage and depth of retained contigs were determined using BWA-MEM2 and SAMtools. For subsequent analyses, retained contigs were considered present within a sample if at least 25% of the bases in the contigs had at least one read mapping to them. In this way, the impact of spurious mappings occurring across coassembled samples (e.g., due to highly conserved regions) is limited. Eukaryotic contigs in metagenome assemblies were identified using EUKsemble (see Section 3.1.1), a hybrid reference and *k*-mer-based approach using contigs with minimum contig lengths of 1 and 3 kbp, respectively. The fractions of eukaryotic, prokaryotic, viral, and unclassified contigs within a metagenome were estimated from the coverage information previously estimated. To capture the diversity within each group, the dissimilarity between the contigs present in each sample was estimated by Mash v2.3^66^ using 20,000 randomly sampled contigs within each sample. For each group, Mash was also used to estimate the β diversity across DWDSs. Before β diversity estimation, assemblies with fewer than 250 contigs were removed, rarefying the remaining assemblies to an equal number of contigs. The existence of significant presence-absence based co-occurrence patterns of 18S rRNA genes was evaluated using CoNet v1.1.1^67^ and Cytoscape v3.9.1^68^ using a hypergeometric distribution-based approach and a significance threshold of 0.05. The obtained network was divided into modules maximizing modularity using the Leiden algorithm^69^ implemented in leidenbase v0.1.12.^70^ The 18S rRNA gene sequence percentage identity of genes within each network module was estimated using blastn as carried out by Wu and collaborators.^71^

#### 2.2.3 Statistical Analyses

Statistical analyses were conducted in R v4.2.1.^72^ Samples were clustered using the *k*-means algorithm based on Nonpareil coverage and logit-transformed eukaryotic bp fraction after the normalization of the two variables. α diversity analyses of SSU rRNA genes were performed using breakaway v4.7.6^73^ and DivNet v0.4.0^74^ modeling the effect on samples of all of the categorical factors (i.e., DWDS of origin and abundance cluster membership), while the correlations among eukaryotic and prokaryotic or viral β diversities were tested using a Mantel test, as implemented in vegan v2.6-2.^75^ Linear mixed-effects models from the lme4 v1.1-29 package^76^ were used to evaluate the differences in water quality characteristics among different clusters using a random effect for accounting for the differences among DWDS. Log transformations were used to correct for residuals’ heteroscedasticity. For each rRNA genes module identified with the network analysis, a hurdle negative binomial model (package countreg v0.2-1^77^) was used to model the number of 18S rRNA genes detected in each sample belonging to the considered module as a function of the disinfection strategy, the source water origin, and the Koppen climate zone^78^ to identify their effect on module detection (i.e., the detection of at least one taxa belonging to the module) and the number of members detected within each module.

## 3 Results and Discussion

### 3.1 Bioinformatic Tool Benchmarking

#### 3.1.1 Eukaryotic Identification

**Figure 1:**
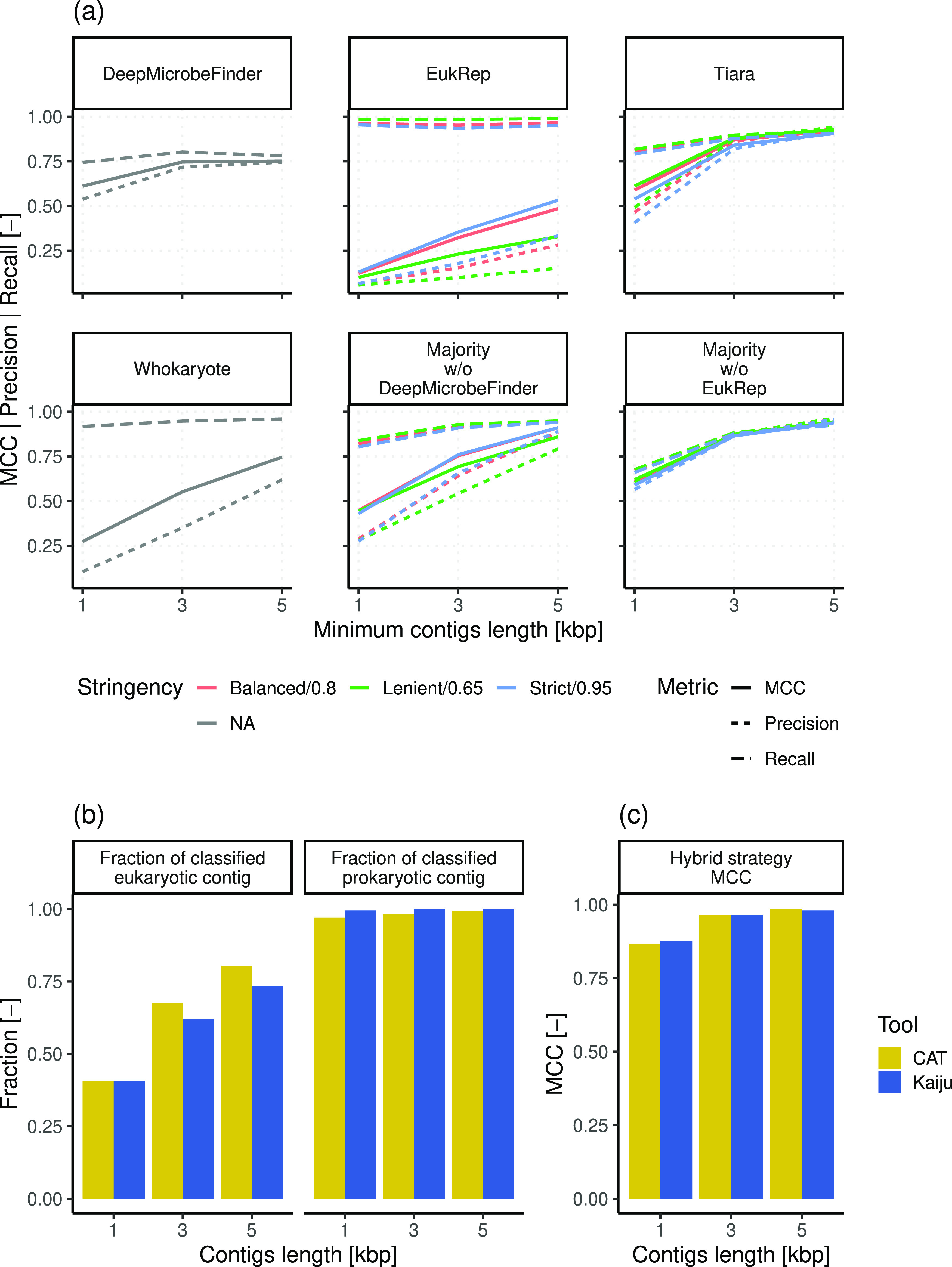
(a) MCC, precision, and recall of tested *k*-mer-based strategies for eukaryotic identification. The stringency levels tested refer to EukRep’s setting and Tiara’s probability threshold used, while NA indicates tools where stringency was not adjustable. (b) Fraction of eukaryotic and prokaryotic contigs identified by reference-based tools and eukaryotic identification MCC. (c) Eukaryotic identification MCC of hybrid *k*-mer- and reference-based strategies. Unitless axis metrics are marked as “[−]”.

The majority of the sequenced data in metagenomic assemblies from complex environmental samples are typically contained in short contigs (e.g., <5 kbp), especially in the case of complex and highly diverse communities with low abundance organisms.^21,79,80^ However, eukaryotic sequence identification tool benchmarks often focus predominantly on longer contigs,^26−29^ potentially leading to overestimating the tools’ performances in complex metagenomes. Eukaryotic sequence identification from metagenome assemblies utilized either *k*-mer signature differences between eukaryotes and prokaryotes or a comparison of unknown sequences with reference databases. As described in previous studies, the performance of *k*-mer-based strategies improves with increasing contig length (Figure 1a). In our benchmark, EukRep resulted in poorer performances compared to the other tools due to the very liberal eukaryotic classification regardless of the settings used, which is consistent with previous results.^26−28^ While this may ensure the recovery of most eukaryotic sequences,^29^ this also might result in higher contamination if a thorough contamination removal step is not performed. Instead, in contrast with recent reports,^28^ Tiara outperformed Whokaryote. This is because the distributions of the gene structure metrics used by Whokaryote depend on contig length, and they are, thus, not generalizable (Figure S3). In fact, while the inclusion of such metrics alongside Tiara’s predictions, as done by Whokaryote, leads to a more accurate classification of long contigs,^28^ their inclusion with short contigs is not effective, likely due to the presence of incomplete and fragmented genes.

Compared to the other tools, DeepMicrobeFinder was trained on short contigs (i.e., 0.5–5 kbp).^26^ This resulted in relatively high MCC values at 1 kbp but led to only a limited improvement with longer contigs, reaching a plateau of its MCC value at 3 kbp (Figure 1a). As the various tools present different strengths and weaknesses,^28^ we tested the performance of majority voting strategies (ties excluded) using either the combination of EukRep, Whokaryote, and Tiara (Majority w/o DeepMicrobeFinder) or DeepMicrobeFinder, Whokaryote, and Tiara (Majority w/o EukRep) against Tiara, the best-performing single tool. The inclusion of EukRep alongside Tiara and Whokaryote (i.e., Majority w/o DeepMicrobeFinder) resulted in lower performances compared to Tiara due to the low precision of both EukRep and Whokaryote. Instead, the use of DeepMicrobeFinder, Tiara, and Whokaryote (i.e., Majority w/o EukRep) resulted in MCC values approximately 3% greater than Tiara due to an increase in precision obtained at the cost of a drop in recall. Noticeably, the larger MCC improvement between 3 and 5 kbp obtained by Majority w/o EukRep (8%) compared to Tiara (6%) suggests further improvements over Tiara with longer contigs.

In addition to *k*-mer-based tools, two reference-based tools were tested (Figure 1b). In contrast to *k*-mer-based approaches, both Kaiju and CAT presented MCC values above 0.99 for 1 kbp long contigs, in concordance with previous benchmarks.^32^ However, this high MCC was associated with the loss of large fractions of eukaryotic contigs that were not classified, especially for shorter contigs; this was likely because of the presence of incomplete fragmented genes.^81^ We further tested the integration of the two approaches to combine the ability to classify all sequences of *k*-mer-based tools with the high-accuracy of reference-based approaches. Such a hybrid strategy classifies contigs primarily using reference-based predictions, resorting to *k*-mer-based results if no reference-based annotation is available. The integration of reference-based strategies with Majority w/o EukRep improved overall performance compared to the use of exclusive reference- or *k*-mer-based strategies, regardless of the reference-based tool used (Figure 1c). In fact, CAT provided a 0.5% higher MCC value than Kaiju with 5 kbp long contigs, while Kaiju resulted in a 1.3% higher MCC value with 1 kbp contigs. This ensemble approach for the identification of eukaryotic sequences from metagenomic data is documented as a workflow, EUKsemble (https://github.com/mgabriell1/EUKsemble). This workflow combines the results of Majority w/o EukRep with Kaiju’s or CAT’s prediction to improve eukaryotic sequence retrieval from metagenomic assemblies. As the combination between *k*-mer-based strategies and CAT or Kaiju leads to similar results, the choice between the two is left to the user, allowing the use of Kaiju in case if the available computing resources are limited.^32^ Noticeably, to maximize eukaryotic retrieval while minimizing false positives, different minimum contig lengths for the two classification strategies can be exploited. For instance, the chosen reference-based tool could be applied to very short contig lengths (e.g., 1 kbp) to retrieve with high confidence as many eukaryotic contigs as possible, while Majority w/o EukRep could be used with contigs longer than 3 kbp, the length at which such a strategy provides satisfactory performance.

#### 3.1.2 Recovering Eukaryotic Metagenome-Assembled Genomes from Metagenomic Assemblies

Previously published eukaryotic-targeted metagenome pipelines rely prevalently on MetaBAT2 or CONCOCT binning directly on the whole metagenome or eukaryotic-screened contigs.^15,29,82^ However, currently, no direct comparison between the various alternatives is present. Hence, we tested the performance of several state-of-the-art binning tools on in silico mock metagenomes either after the selection of eukaryotic contigs (EUK-only) or directly on the whole metagenome (FULL) using a range of minimum contig lengths. As eukaryotic identification was performed using EUKsemble with different minimum contig lengths for *k*-mer- and Kaiju-based identification (i.e., 3 and 1 kbp), we also tested the possibility of performing binning with contigs identified as eukaryotic or without any assigned superkingdom (OTHER-rem) after the removal of only the contigs classified as noneukaryotic (i.e., prokaryotic, viral).

**Figure 2:**
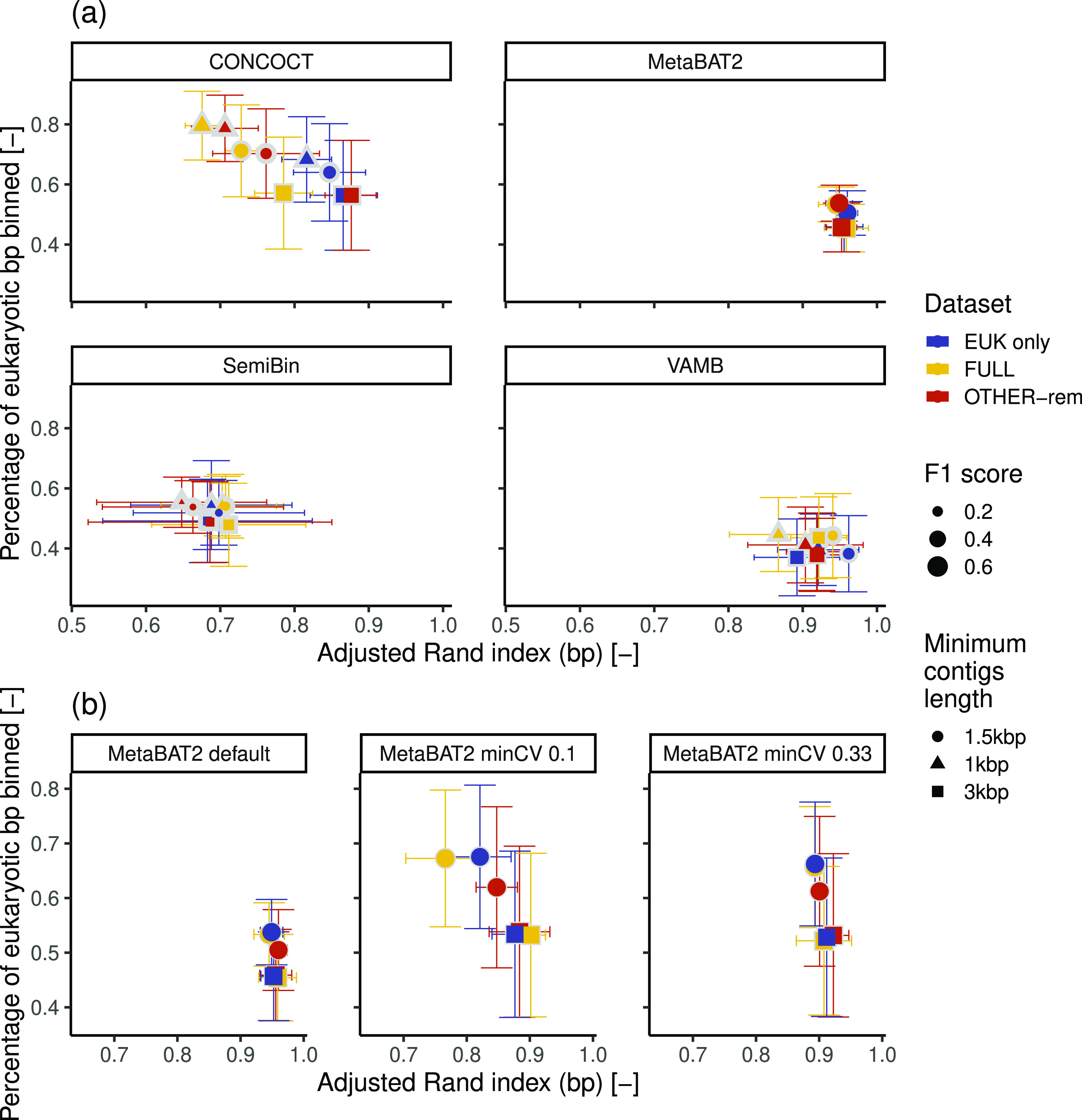
(a) Average and standard deviation of the fraction of eukaryotic bp binned and ARI obtained by the tested binners on the simulated metagenomes. (b) Effect of the variation of the MetaBAT2’s parameter minCV on the percentage of eukaryotic bp binned and ARI. In both (a) and (b), the size of the outer marker depicted in light gray represents the *F*1 score estimated using only the most complete bin per each eukaryotic genome recovered, while the size of the inner marker represents the value including all generated bins.

Binning tools suffered inherently from a tradeoff between the amount of assembled bp included in the recovered bins and the binning quality (Figure 2a and Table S4), as reported by similar benchmarks.^83^ Our results indicate that this is observable both across different tools, minimum contig lengths, and binning strategies (Figure 2a). CONCOCT generally recruits the most eukaryotic bp into bins, while, conversely, MetaBAT2 and VAMB maximize the ARI, indicating higher quality of the reconstructed eukaryotic bins. SemiBin performs poorly on both metrics. Increasing the minimum contig length thresholds, on the one hand, improves the bin quality for all binning tools (i.e., higher ARI values) while coincidentally resulting in lower fractions of eukaryotic bp within bins. Direct binning of the entire metagenome allows recovery of a higher fraction of eukaryotic bp present in the metagenome but at the cost of a lower ARI as compared to binning exclusively on contigs annotated as eukaryotic. The limited eukaryotic recovery arises due to the limits of reference-based eukaryotic identification, which do not classify a large fraction of the eukaryotic contigs between 1 and 3 kbp (Figure 1b). In contrast, excluding only the contigs classified as noneukaryotic provided an increase in the ARI value for all binners except for SemiBin and especially using CONCOCT (average percentage increase: CONCOCT, 7%; MetaBAT2, 1%; VAMB, 1%) compared to binning the entire metagenome while recovering up to 15% more bp than binning exclusively contigs annotated as eukaryotic. The selection between binning only the contigs identified as eukaryotic or including also those nonclassified can depend on the acceptable level of contamination of the recovered bins. Including nonclassified contigs may require extensive curation (e.g., Delmont and collaborators^84^) as this practice can result in highly chimeric bins including both eukaryotic and prokaryotic contigs (Figure S4) that are likely to affect downstream results.

As MetaBAT2 (with default settings) resulted in the highest ARI, we tested whether it was possible to increase the recovery of eukaryotic data by including contigs with low coverage depths. Indeed, as shown in Figure 2b, reducing the value of minCV, the parameter controlling the minimum coverage depth admissible, increased the recovery, reaching values comparable to CONCOCT. However, excessively low minCV values (i.e., 0.1) affected ARI values negatively, indicating the value of 0.33 as a suitable lower bound. The need to adjust this parameter does not require prior knowledge of the microbial community analyzed but only a previous identification of eukaryotic contigs and coverage depth estimation. The highest average *F*1 scores of the most complete bin per recovered genome were provided by MetaBAT2 with reduced minCV parameter values and CONCOCT, followed by default MetaBAT2, SemiBin, and VAMB, mostly due to the variations in completeness, as purity showed high average values (i.e., >0.9) (Table S4). However, when considering all of the recovered bins, MetaBAT2 led to the highest scores since the other binners show high fragmentation of the initial genomes. This fragmentation was not associated with the chromosomal organization of eukaryotic genomes but rather due to the combination of high *k*-mer diversity and low coverage depth (Figure S5, Meyer and collaborators^21^). Both the eukaryotic identification and binning benchmark results highlight how the length of the assembled contigs plays a significant role in eukaryotic recovery from metagenomes. Even though a systematic benchmark is needed, these results suggest metaSPAdes coassembly as the most appropriate assembler to recover eukaryotes from metagenomes, being known to produce longer contigs than other assemblers and single-sample assembly strategies.^21,80,85^ As the DWDS microbiome is largely unexplored, the results of these benchmarks will aid future studies dedicated to the characterization of the eukaryotic communities present in drinking water systems, where issues linked to the low relative abundance of eukaryotes are expected.

### 3.2 Factors Affecting Eukaryotic Relative Abundance in DWDS Metagenomes

**Figure 3:**
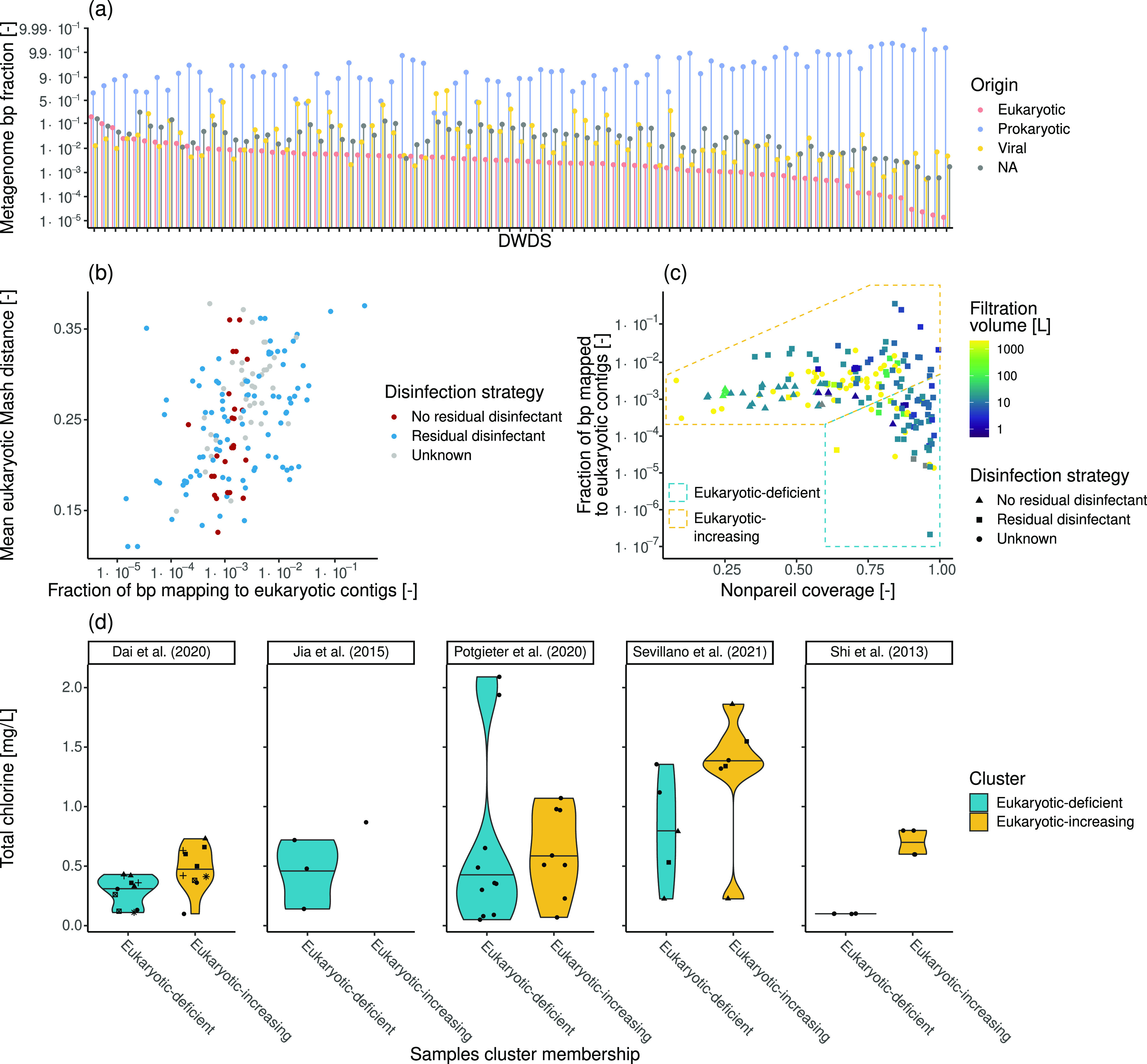
(a) Fractions of eukaryotic, prokaryotic, viral, and not-classified (NA) bp in each of the analyzed metagenomes. EUKsemble results refinement is based on the classification provided by Kaiju. (b) Association between the fraction of bp mapping to eukaryotic contigs and the mean Mash distance between eukaryotic contigs in each sample as a function of the disinfection strategy employed. (c) Relationship between the Nonpareil coverage and the fraction of bp mapping to eukaryotic contigs in each sample. (d) Total chlorine concentrations from DWDSs whose samples belong to both the eukaryotic-deficient and eukaryotic-increasing clusters. The different shapes in each facet in panel (d) indicate different DWDSs. A logit scale was applied in panels (a)–(c) to improve the clarity of the presentation.

The eukaryotic fraction of DWDS metagenomes was assessed on a total of 181 samples collected from 81 DWDSs across the globe (Figure 3a) using EUKsemble relying on Kaiju’s reference-based approach. Even though a few studies showed particularly high fractions of bp mapping to eukaryotic contigs, most DWDSs showed fractions of eukaryotic bp below 1%. Such low amounts of recovered bp, being in some cases shorter than the genome of a single eukaryotic genome,^86^ are similar to previous results^35,55,87^ and prevented MAG reconstruction. In fact, despite the intensive eukaryotic identification procedure, eukaryotic contigs presented, in most cases, lower fractions than those mapping to viral contigs identified based on Kaiju’s classification. Still, the retrieved eukaryotic percentages are likely underestimated due to the limits of eukaryotic identification and the exclusion of very short contigs (<1 kbp) because of their limited reliability in further analyses, such as binning.^88^ The presence of a good correlation between the recovered eukaryotic bp and the 18S rRNA genes identified suggests that our workflow is effective at recovering the majority of the eukaryotic bp in the investigated metagenomes, confirming the relative abundance trend observed for eukaryotes (Figure S6). Future bioinformatics advancements could, however, allow better recovery, further minimizing the fraction of unclassified bp. Despite the possible confounding effect caused by the heterogeneity within the data, higher fractions of assembled and identified eukaryotic bp are associated with higher eukaryotic diversity within samples, especially in disinfected systems where most data is available (Figure 3b; Spearman correlation disinfected systems, 0.48; *p*-value, <0.001; nondisinfected systems, 0.35; *p*-value, 0.081). This result further suggests that eukaryotic populations are systematically undersampled using current metagenomic approaches and is in line with the trends for viruses recovered in nondisinfected systems (Figure S7) while being in contrast with prokaryotes for which the available data showed no significant relationship between their bp fraction and their diversity (Figure S8).

Besides the variability of eukaryotic relative abundances in the investigated DWDSs, differences in the recovery of eukaryotic DNA in metagenomes could be due to the different experimental protocols used in the various studies (Table S3), ranging from sample collection to sequencing strategies used. Common filter size for microbial concentration ranges from 0.2 to 0.45 μm and should not affect eukaryotic recovery.^89^ The eukaryotic metagenome fractions do not correlate with the filtered water volume (*p*-value = 0.69) reported in corresponding studies (Figure 3c), indicating that filtering a larger volume of water does not improve eukaryotic recovery in metagenomes. Specifically, while filtering larger volumes may increase the number of eukaryotic cells captured, their ratio relative to prokaryotes and viruses will not change and thus may not result in greater recovery of eukaryotic sequences in metagenomes. DNA extraction prior to metagenomic analysis can also affect the microbial community recovered using metagenomic sequencing strategies.^90,91^ However, as samples taken from the same DWDSs were extracted using the same extraction method per DWDS, it was not possible to assess the effect of this factor. In any case, while commercial extraction methods were shown to be able to successfully extract eukaryotic DNA,^92,93^ specific processing techniques^94^ or the use of dedicated enzymes^95^ might further increase yields and/or quality. However, the variety of eukaryotic phenotypes (e.g., soft-shelled, hard-shelled) exacerbates the extraction bias, making it unlikely that a single optimal extraction method could be developed.^96^ At last, the sequencing depth affects the ability to recover rarer taxa.^97^ However, increased sequencing efforts did not lead to a significant increase in eukaryotic fractions (*p*-value = 0.38) due to the confounding effect caused by the different complexity of the microbial community in each sample.^98^ Indeed, irrespective of the actual sequencing depth, better characterization of the microbial community, as indicated by higher Nonpareil coverage,^61^ allows higher eukaryotic fractions in metagenomes (Figure 3c), providing evidence of the underestimation of eukaryotic presence in DWDSs. Given this result, together with the fact that most previous studies have focused on prokaryotes,^99^ it is likely that the role of eukaryotes in shaping the microbiome of drinking water systems is currently underappreciated.

Despite exhibiting high Nonpareil coverages, some samples show extremely low eukaryotic fractions, separating an “eukaryotic-deficient” cluster from the “eukaryotic-increasing” one (Figure S9); this includes samples originating from the same DWDS splitting into these two clusters. The two clusters show a significantly different composition with respect to source water type (χ^2^ test, *p*-value <0.001), with the eukaryotic-deficient cluster enriched in samples derived from groundwater-fed systems (23%) compared to the eukaryotic-increasing cluster (5%), suggesting higher eukaryotic relative abundances in surface water-fed systems. This is in concordance with what was previously observed in raw waters.^100^ However, samples from surface water-fed DWDSs were abundant in both clusters (eukaryotic-deficient cluster = 42.6%, eukaryotic-increasing cluster = 29.2%). Water disinfection is an important factor affecting the drinking water microbiome.^35,99^ In fact, when comparing the total chlorine concentrations in samples obtained from the same DWDS but belonging to different clusters, eukaryotic-deficient samples presented lower chlorine concentrations (95% confidence interval, −195 to −29%; Figure 3d). Eukaryotes typically have higher resistance to disinfectants compared to bacteria,^6,101,102^ and thus, the higher chlorine concentrations present in samples belonging to the eukaryotic-increasing cluster could have altered the relative abundances, leading to a higher eukaryotic DNA recovery in the metagenomes, similar to what was observed by Dai and collaborators.^35^ In fact, higher chlorine concentrations might limit prokaryotic growth within DWDSs despite the presence of available nutrients^103^ and maintain abundances similar to water treatment outlets. On the other hand, several countries limit prokaryotic growth by reducing the nutrients available (i.e., carbon, nitrogen, etc.) in finished drinking water.^103^ Indeed, several samples with low Nonpareil coverages from nondisinfected systems are included in the eukaryotic-increasing cluster, suggesting that limiting microbial growth may be associated with enhanced eukaryotic detection in metagenomes. This consideration, coupled with the results presented in Figure 3d, highlights the importance and the interaction of the multiple stresses (i.e., disinfection and nutrient limitation) in shaping drinking water microbiology. In fact, as such stresses are used to limit excessive microbial growth, insights into their effects and interaction are critical for DWDS microbiome management. Finally, samples belonging to the eukaryotic-deficient cluster present lower average estimated eukaryotic richness (*p*-value = 0.002) and nonsignificant differences in Simpson and Shannon diversities (*p*-values >0.28) compared to the samples belonging to the eukaryotic-increasing cluster at similar Nonpareil coverages (i.e., >0.75), indicating the presence of less diverse and more even communities. Besides water sources and DWDS management strategies, these observations could be associated with other factors that could not be included in this study due to the lack of this information. For example, water treatments, water chemistry, and location within DWDSs have been shown not just to affect prokaryotic but also eukaryotic abundances^6,104,105^ and should be the focus of targeted studies.

### 3.3 Factor Affecting Eukaryotic Diversity in DWDS Metagenomes

**Figure 4:**
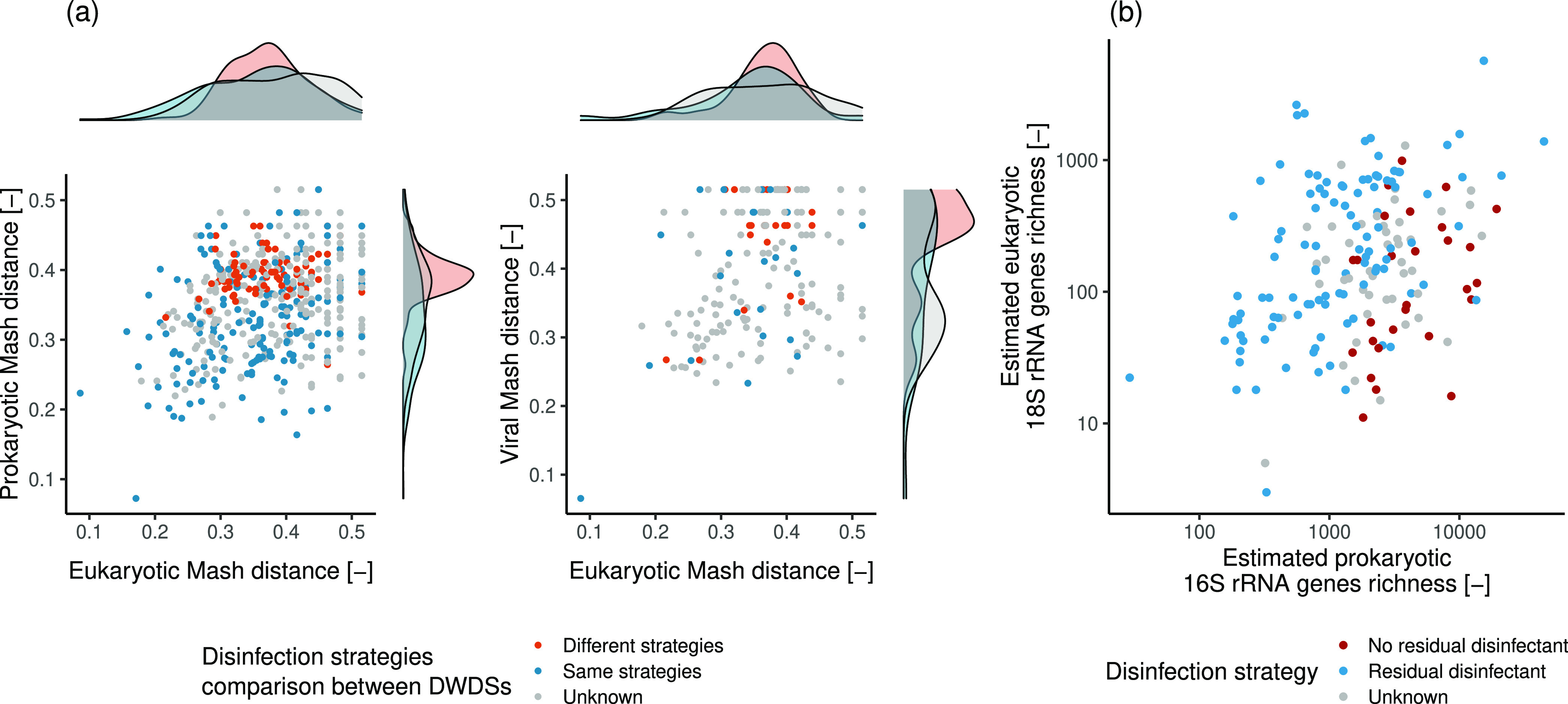
(a) Eukaryotic, prokaryotic, and viral β diversity correlations and marginal distributions as a function of the sample disinfection strategy. (b) Estimated richness of eukaryotic and prokaryotic rRNA genes with respect to the disinfection strategy. Log scale axes were used in panel (b) to improve clarity.

As environmental factors and DWDS management strategies affect the proportion of eukaryotes, prokaryotes, and viruses in DWDS metagenomes, these factors could also affect the taxa present and the diversity across DWDSs. In fact, eukaryotic β diversity correlates positively with those of both prokaryotes and viruses (Figure 4a, eukaryotic–prokaryotic Mantel statistic *r* = 0.25, *p*-value = 0.002; eukaryotic–viral Mantel statistic *r* = 0.21, *p*-value = 0.014). While such low values are likely caused by the heterogeneity of upstream treatments and water conditions in the various studies, correlations among β diversities suggest that spatiotemporal dynamics and factors that were found to influence prokaryotes and viruses (e.g., disinfection strategies, seasonality, water age),^103^ are likely to be relevant also for eukaryotes. Such concordance is likely the result of both direct causes affecting both eukaryotes and other taxonomic groups (e.g., upstream water treatment, nutrient availability, disinfection stress)^7,104^ or could arise indirectly as a result of their interactions. In fact, depending on environmental stresses (i.e., nutrient availability), fungi have been shown to modulate bacterial growth levels,^106^ while protists can both host and eventually select specific prokaryotic symbionts and viruses, favoring their multiplication,^107−109^ and selectively predate on them,^110,111^ highlighting the role of eukaryotes in shaping microbiomes. In fact, specific eukaryotes could potentially be used to develop ecologically-informed management strategies relying, for example, on their predation of selected harmful microorganisms^112^ or their alteration of biofilm structure, minimizing biofouling.^113^

Through the analysis of the 18S and 16S rRNA genes, it was possible to show a positive correlation between the estimated eukaryotic and prokaryotic richness (Figure 4b; disinfected systems = 0.5, *p*-value < 0.001; nondisinfected systems = 0.36, *p*-value = 0.046). The presence of such a correlation, also observed by Yeh and Fuhrman,^114^ is concordant with the “diversity begets diversity” hypothesis,^115^ likely arising due to the interactions between populations across superkingdoms,^6^ which expand the availability of ecological niches and thus enhance diversity. Figure 4a,b further underlines the effect of disinfection on the DWDS microbiome, highlighting, in accordance with Dai^35^ and Hegarty^116^ and collaborators, the effect of disinfection strategies on the β diversity of prokaryotic and viral communities in DWDSs and suggesting a lower effect for eukaryotes (median Mash differences: eukaryotes = 0.021; prokaryotes = 0.068; viruses = 0.043). While this result is concordant with the higher chlorine resistance of eukaryotes,^6,101,102^ it should be noted that given the likely undersampling of eukaryotic communities in DWDSs, such result might be biased toward the most abundant eukaryotes and that further dedicated studies would be needed to confirm it.

**Figure 5:**
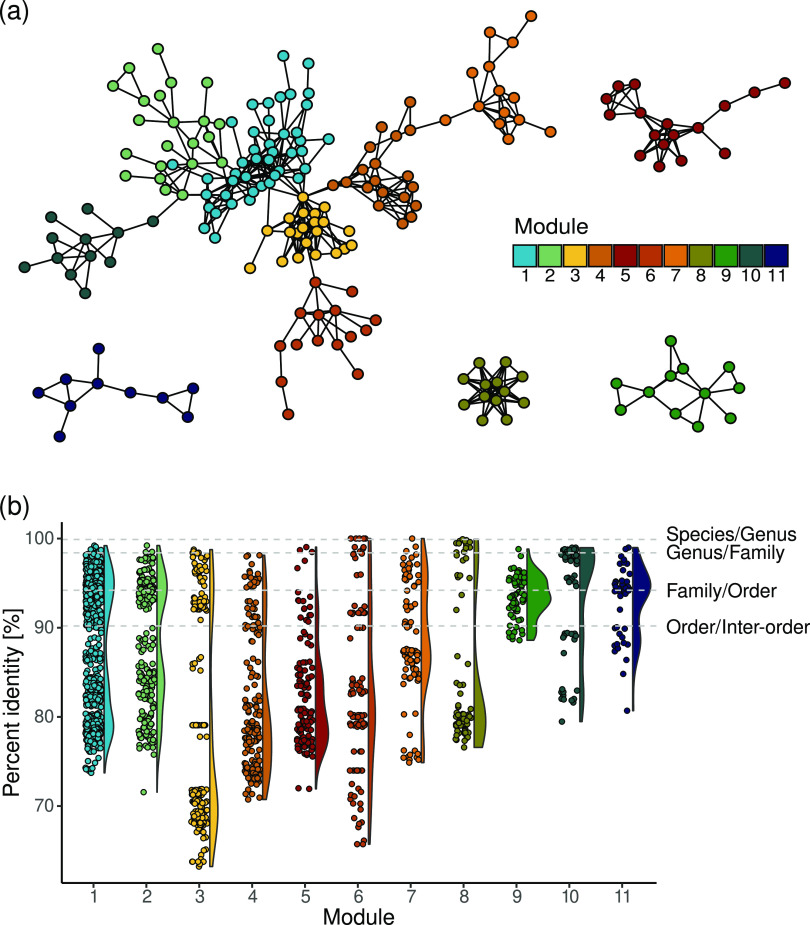
(a) Network of co-occurring eukaryotic 18S rRNA genes colored by the module. (b) Pairwise similarity of 18S rRNA genes expressed as percent identity (%) within each module.

The presence-absence-based co-occurrence analyses of the 18S rRNA genes present in the samples was carried out to obtain more insights into the eukaryotic communities in the DWDS metagenomes. This approach was favored compared to relative abundance-based co-occurrence analyses to limit the confounding effects caused by the different experimental protocols employed in the different studies. This network analysis indicated the presence of 11 18S rRNA gene modules, each composed of more than 10 eukaryotic taxa (Figure 5a). The 18S rRNA gene sequence similarity analyses within each module indicated that between 13 and 86% of the genes in each module belong to taxa within the same order,^71^ with several members belonging to the same family or genus (Figure 5b), as also observed in the cluster members’ taxonomy reported (Table S5). The variation in the ranges of percentage identity distributions and the shape of the density distributions suggest the presence in each module of different groups of phylogenetically similar taxa with different degrees of phylogenetic relatedness, possibly arising from several evolutionary and ecological factors.^117^ It is important to note that the co-occurrence patterns retrieved here do not necessarily confirm ecological interactions^118^ and should be confirmed by further hypothesis-driven studies. This is especially valid for phagotrophic organisms, while less so for eukaryotes that can feed on other eukaryotes such as nematodes.^119^ Noteworthily, nematodes make up most of the nodes in modules 4, 7, and 9. While little information on their diet in drinking water systems is available, some studies in other environmental matrices report that certain species are known to prey on the same, possibly eukaryotic, microorganisms (i.e., fungal-feeder *Aphelenchoides spp.*, present in module 4) or even other nematodes (i.e., genus *Mesodorylaimus*, present in module 4), possibly explaining the associations found.^119,120^ In addition, some of the co-occurrences retrieved that involve parasitic nematodes are possibly due to the infection of similar hosts, as the plant parasites *Longidorus* spp. and *Xiphinema* spp.^121^ (both present in module 4).

**Figure 6:**
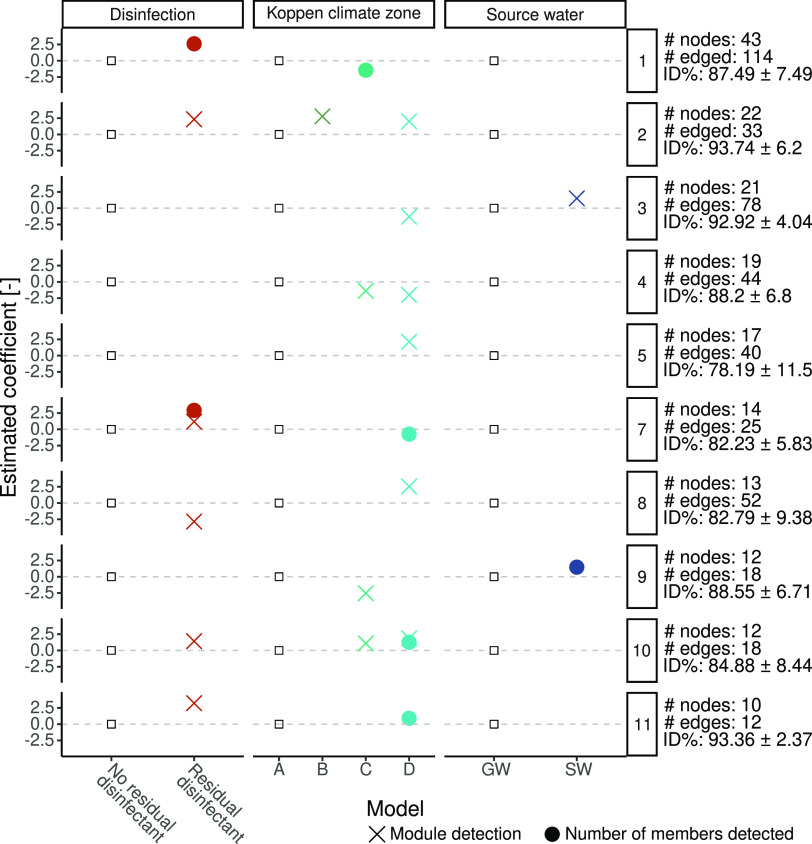
Estimated hurdle negative binomial models coefficients for module detection and number of members detected as a function of the disinfection strategy, climate zone, and source water type. The coefficients are to be interpreted as relative effects compared to the factors marked with a white square. Neither the factor “Unknown” nor the modules with no significant predictors are shown. Information on the right side reports the number of nodes and edges within each module and the average and standard deviation of the pairwise 18S rRNA genes sequence identities within each module.

The detected association could be considered as groups of eukaryotes that present similar responses to environmental and DWDS management factors or even other (micro)biota. Such interpretation is supported by the analysis, within each module, of the number of members detected as a function of the DWDS disinfection strategy, climate zone, and source water origin. Except for module 6, which did not show any significant predictors (i.e., *p*-value < 0.05) of its detection or the number of its members detected, all of the modules showed variations due to the tested factors (Figure 6). While the higher detections for some modules and module members in disinfected systems might be due to the generically higher Nonpareil coverage of samples derived from such systems, noticeably, module 8 shows lower detection in disinfected systems, indicating potentially the higher sensitivity of its members to disinfection or the adaptation to low-nutrient conditions of nondisinfected DWDSs. In fact, some nodes included in module 8 represent fungi for which some species are known to proliferate under oligotrophic conditions^122^ and demonstrate higher chlorine resistance than viruses and prokaryotes but lower than protists cysts and oocysts.^101,123^ In accordance with the expected climatic differences and geographical distances among the two zones,^78^ climate zone D (i.e., continental) showed, in most cases, differences in the detection of the module members compared to zone A (i.e., tropical). Finally, in accordance with the results of Section 3.2, higher detection was observed in drinking water produced from surface water for selected modules. For example, module 3 is composed mostly of Eustigmatophyceae, a lineage of photosynthetic algae present in freshwater,^124^ indicating the possible role of the eukaryotes (and/or their genetic material) in source waters in seeding downstream DWDSs. Besides the factors taken into account in this analysis, it is important to note that several other factors might have affected the detection of modules and module members (e.g., upstream treatment, physicochemical water quality, degree of eukaryotic community characterization). Future analyses considering such parameters will shed further information on the factors affecting the eukaryotes within DWDSs, opening new opportunities for their management. Nonetheless, the results provided can already help water utilities to assess which eukaryotes are most likely to be present within their DWDSs and, in case of the presence of microbial quality issues, plan appropriate interventions.

## 4 Implications for Future Research and Drinking Water Systems

The results of this study highlight the under-representation of eukaryotes within current DWDS metagenomes. To accurately determine the relative (or absolute) abundance of eukaryotes and the membership and structure eukaryotic communities within the drinking water microbiome, sampling protocols and extraction methods should be adapted to enrich for eukaryotic microorganisms, as already done in different fields surveys, where sampling and laboratory techniques are tailored depending on the microorganisms of interest. For example, laboratory protocols used in the Tara Oceans Expedition were either carefully selected among existing ones or specifically developed to limit potential biases and ensure the quality and comparability of the results.^94^ Furthermore, this expedition applied a comprehensive sampling strategy that selected different microorganisms using a size-fractionation approach based on previously available data.^125^ Finally, a wide set of environmental conditions was also collected to aid data interpretation.^126^ In the drinking water field, a similar standardized initiative was carried out in The Netherlands to monitor macroscopic invertebrates using optimized sampling techniques and microscopic techniques,^127^ but it is not yet widely adopted.

Despite the recent growing attention, eukaryotic-focused metagenomics is not as well established compared to the prokaryotic or viral counterparts, with studies reporting its complexity with current approaches.^22^ Likely, the combination of multiple strategies, as done in EUKsemble, and the use of novel approaches, such as dedicated assembly workflows and the further exploitation of assembly graph information,^128^ would enable improvements in eukaryotic-focused metagenomics. For example, a novel pipeline for eukaryotic gene calling combining several previous tools has shown improved performances with respect to previous methods, allowing its use for the analysis of large-scale data.^129^ Given the importance of contigs length on both eukaryotic identification and binning, the use of accurate long-reads sequencing would likely be highly beneficial for both these tasks, also providing the opportunity to recover full-length 18S rRNA genes to populate reference databases.^130^ Compared to prokaryotes and viruses, both the reference data and the option of tools available for eukaryotes are limited, further exacerbating the complexity of the reconstruction of their genomes. In addition, due to the wealth of data provided by marine expeditions, reference databases included in several tools are highly skewed toward marine taxa (e.g., Levy Karin and collaborators,^81^ Vaulot and collaborators^131^), potentially limiting and biasing the analyses performed on other environments. While new tools will improve the analysis of eukaryotes from mixed metagenomes, only focused sampling efforts are needed to overcome the compositional bias of current references and enable a clearer view of eukaryotes in DWDSs.

Our results highlight that eukaryotes are present at low relative abundances in DWDSs worldwide. While some of the taxa found, including heterotrophic and mixotrophic microorganisms such as protists, fungi, and metazoan, are frequently detected in DWDSs, others, such as strictly photosynthetic algae, are likely to be present only due to their breakthrough (or that of their genetic material) of upstream water treatments, especially in the case of DWDSs fed by surface water where relative eukaryotic abundance is higher. These microorganisms, although unable to grow in DWDSs, can represent a possible substrate source for the necrotrophic growth of other eukaryotic and prokaryotic microorganisms,^132^ potentially limiting the effectiveness of substrate removal efforts, and cause taste and odor issues.^133^ In fact, the presence of both single and multicellular eukaryotes and the identified positive diversity and richness correlations support the presence of a complex food web within DWDSs where eukaryotes could be both predators of prokaryotes and viruses^110,111^ but also be prey of other eukaryotes^134^ or hosts of other taxa.^135^ As a result, besides direct management strategies affecting all microorganisms and viruses in DWDS (e.g., disinfection), management strategies targeting specific taxa could indirectly affect other potentially detrimental taxa, such as opportunistic pathogens,^112^ or impact unrelated operational issues, such as water discoloration.^10^

A better understanding of the ecological role of eukaryotes in DWDSs provided by both experimental and bioinformatic advancements deepens our understanding of current microbiological management strategies in DWDSs (e.g., disinfection and nutrient starvation). Such information could be used to not only limit the presence of unwanted microorganisms (e.g., Cavallaro and collaborators^112^) but also to devise new ecologically informed microbiological management plans, improving both water treatment and distribution (e.g., Derlon and collaborators^113^).

## Supporting Information Available

Detailed data regarding the bioinformatic tools benchmarking, the samples used in the analyses, and the nodes in the co-occurrence network (XLSX)
Eukaryotic identification benchmarking workflow for *k*-mer-based, reference-based, and hybrid strategies (Figure S1); eukaryotic binning benchmarking workflow (Figure S2); normalized intersection of the histograms of Whokaryote predictors and modal bin values of the histograms of the predictors for eukaryotic and prokaryotic contigs (Figure S3); cross-superkingdom contamination of the bins recovered by the tested binning algorithms (Figure S4); fragmentation of eukaryotic genomes (Figure S5); number of eukaryotic SSU (i.e., 18S) rRNA genes identified from the cleaned reads compared to the bp mapped to the contigs identified as eukaryotic (Figure S6); viral MASH distance and prokaryotic Mash distance as a function of the viral and prokaryotic fractions of the metagenomes investigated (Figures S7 and S8, respectively); and samples’ probability density as a function of Nonpareil coverage and fraction of eukaryotic bp with respect to the boundaries of the identified clusters (Figure S9) (PDF)
